# Predictors of clinically relevant postoperative morbidity following curative intent surgical resection for non-small cell lung cancer

**DOI:** 10.25122/jml-2026-0063

**Published:** 2026-04

**Authors:** Cosmin Bogdan Tanase, Elena Chitoran, Vlad Rotaru, Giuseppe Gullo, Teodor Horvat, Laurentiu Simion

**Affiliations:** 1 Carol Davila University of Medicine and Pharmacy, Bucharest, Romania; 2 Bucharest Institute of Oncology Prof. Dr. Alexandru Trestioreanu, Bucharest, Romania; 3 Villa Sofia Cervello Hospital, University of Palermo, Palermo, Italy

**Keywords:** non-small cell lung cancer, postoperative morbidity, thoracic surgery, video-assisted thoracoscopic surgery, postoperative complications, Clavien–Dindo classification, VATS, video-assisted thoracoscopic surgery, NSCLC, non-small cell lung cancer, FEV1, forced expiratory volume in 1 second, CT, computed tomography, PET-CT, positron emission tomography-computed tomography, ASA, American Society of Anesthesiologists, CCI, Charlson Comorbidity Index, ICU, intensive care unit, LLL, left lower lobe, LUL, left upper lobe, RLL, right lower lobe, RML, right middle lobe, RUL, right upper lobe, NAT, neoadjuvant therapy

## Abstract

Postoperative morbidity remains an important challenge following curative-intent surgical treatment for non-small cell lung cancer (NSCLC), despite advances in minimally invasive thoracic surgery and perioperative management. Identification of factors associated with clinically relevant postoperative complications may improve perioperative risk stratification and patient selection. The aim of the present study was to evaluate predictors of clinically relevant postoperative morbidity following pulmonary resection for NSCLC in a single tertiary oncologic center. A retrospective observational cohort study was conducted that included 163 consecutive patients who underwent curative-intent anatomical pulmonary resection for NSCLC. Clinically relevant postoperative morbidity was defined as Clavien–Dindo grade ≥ II. Demographic, clinical, functional, and perioperative variables were analyzed, including age, Charlson Comorbidity Index (CCI), pulmonary function (FEV1), surgical approach, pathological stage, and type of resection. Comparative analysis between patients with and without clinically relevant morbidity was performed. Univariate and multivariate logistic regression analyses were subsequently performed to identify factors associated with postoperative morbidity. Model performance was evaluated using receiver operating characteristic (ROC) curve analysis. Clinically relevant postoperative morbidity occurred in 21.5% of patients. Patients who developed postoperative complications tended to be older and presented lower preoperative FEV1 values compared with patients without clinically relevant morbidity. Increased CCI demonstrated an association with increased postoperative morbidity risk (adjusted OR = 2.04, 95% CI, 0.54–7.70), while lower FEV1 values were associated with increased postoperative risk (adjusted OR = 0.98, 95% CI, 0.95–1.01). Increasing age also demonstrated a modest association with postoperative complications (adjusted OR = 1.02, 95% CI, 0.97–1.07). The final multivariate model demonstrated fair discriminatory performance, with an area under the ROC curve (AUC) of 0.645. Clinically relevant postoperative morbidity following NSCLC surgery appears to be influenced by multiple patient-related and procedure-related factors, particularly comorbidity burden, pulmonary reserve, and age. Although the predictive performance of the present exploratory model was modest, careful preoperative evaluation and individualized perioperative risk stratification remain essential in thoracic oncologic surgery.

## Introduction

Lung cancer remains the leading cause of cancer-related mortality worldwide and continues to represent a major global healthcare burden despite advances in diagnostic and therapeutic strategies [1]. Non-small cell lung cancer (NSCLC) accounts for approximately 85% of all pulmonary malignancies and includes several histopathological subtypes, most commonly adenocarcinoma and squamous cell carcinoma [2]. Surgical resection remains the standard curative-intent treatment for patients with resectable NSCLC and plays a central role in multimodal oncologic management [3].

Over the past two decades, thoracic surgery has undergone a significant transition from conventional thoracotomy toward minimally invasive techniques, particularly video-assisted thoracoscopic surgery (VATS). Minimally invasive approaches have progressively gained acceptance due to their association with reduced postoperative pain, shorter hospitalization, lower intraoperative blood loss, and faster postoperative recovery compared with open surgery [4,5]. Furthermore, several studies have demonstrated comparable oncologic outcomes between VATS and thoracotomy in appropriately selected patients with NSCLC [6,7].

Despite continuous improvements in surgical techniques, perioperative care, and patient selection, postoperative morbidity remains a major concern following pulmonary resection for lung cancer. Postoperative complications are associated with prolonged hospitalization, delayed initiation of adjuvant treatment, increased healthcare costs, and impaired quality of life [8]. In addition, severe postoperative complications may negatively influence both short-term and long-term oncologic outcomes [9].

The occurrence of postoperative morbidity after lung cancer surgery is influenced by multiple patient-related and procedure-related factors. Previous studies have identified advanced age, impaired pulmonary reserve, elevated Charlson Comorbidity Index (CCI), prolonged operative time, and open surgical approach as potential predictors of adverse postoperative outcomes [10–12]. Accurate perioperative risk stratification is therefore essential in thoracic oncology, particularly in patients presenting with significant comorbidities or borderline respiratory function.

The Clavien–Dindo classification system has become one of the most widely accepted methods for standardized reporting of surgical complications and allows objective assessment of postoperative morbidity severity [13]. Clinically relevant complications, particularly those requiring invasive intervention or intensive care management, represent important indicators of perioperative quality and surgical safety.

Although numerous studies have compared minimally invasive and open approaches in NSCLC surgery, many have focused predominantly on survival outcomes, lymph node staging, and oncologic radicality, while predictors of clinically relevant postoperative morbidity remain comparatively underexplored [14,15]. Furthermore, data originating from Eastern European thoracic oncology centers remains relatively limited in the current literature.

The aim of the present study was to evaluate the factors associated with clinically relevant postoperative morbidity in patients undergoing surgical treatment for NSCLC in a single tertiary oncologic center. We specifically aimed to identify variables associated with increased odds of postoperative complications using routinely available demographic, clinical, functional, and perioperative variables.

## Material and Methods

### Study design and patient selection

This study was designed as a retrospective observational cohort analysis conducted at the “Prof. Dr. Alexandru Trestioreanu” Institute of Oncology, Bucharest, Romania. Medical records of patients who underwent surgical treatment for non-small cell lung cancer between January 2016 and December 2023 were retrospectively reviewed.

The study protocol was conducted in accordance with the principles of the Declaration of Helsinki and approved by the local Institutional Ethics Committee (approval number 211/19.12.2024). Due to the retrospective nature of the study, individual informed consent was waived; however, all patients admitted to the institution signed a general consent form allowing the anonymous use of clinical data for scientific and educational purposes.

Patients were considered eligible for inclusion if they met the following criteria: histologically confirmed NSCLC; curative-intent anatomical pulmonary resection (either through open thoracotomy or video-assisted thoracoscopic surgery – VATS); complete perioperative and postoperative medical records available for analysis. Exclusion criteria included: incomplete clinical or histopathological records; non-oncologic pulmonary resections; wedge resections without systematic lymph node dissection; patients with small-cell lung cancer (SCLC).

The final study cohort comprised 163 consecutive patients with pulmonary cancer stages IA-IIIB. All cases were discussed within a multidisciplinary tumor board before surgery. All surgical procedures were performed by experienced thoracic surgeons within the same tertiary oncologic center, in accordance with standard oncologic surgical principles.

### Preoperative evaluation

Demographic and clinical variables collected included: age, sex, exposure to toxic respiratory agents, tumor localization, clinical and pathological TNM stage, and neoadjuvant therapy history.

All patients underwent a standardized preoperative assessment protocol, including clinical examination, pulmonary function testing, thoracic and upper abdominal contrast-enhanced computed tomography (CT), and selective positron emission tomography-computed tomography (PET-CT), with cardiologic evaluation when indicated.

Pulmonary function was assessed using forced expiratory volume in one second (FEV1). Comorbidity burden was quantified using the Charlson Comorbidity Index (CCI), while anesthetic and perioperative risk were evaluated using the American Society of Anesthesiologists (ASA) physical status classification. In this study, patients were stratified into two groups based on comorbidity burden: CCI < 4 (low-to-moderate risk) and CCI ≥ 4 (high-risk), as previously validated in surgical outcome research.

### Surgical procedure

All surgical procedures were performed by experienced thoracic surgeons within the same tertiary oncologic center and aimed to achieve complete oncologic resection with systematic mediastinal lymph node dissection, in accordance with standard oncologic surgical principles. The selection of the surgical approach (VATS versus open thoracotomy) was individualized and based on tumor-related characteristics, patient comorbidity profile, anatomical considerations, and surgeon expertise rather than random allocation. Although the degree of VATS experience varied among surgeons, all participating operators had substantial expertise in thoracic oncologic surgery. All minimally invasive procedures were carried out using a multiport VATS technique.

Data on the type of surgical procedure performed for each patient (open vs. VATS, lobectomy vs. segmentectomy) were recorded.

### Postoperative outcomes

Postoperative adverse events were assessed and categorized using the Clavien–Dindo classification system. Morbidity was classified as clinically relevant if the Clavien-Dindo class was ≥2.

For each patient, postoperative intensive care unit (ICU) stay, overall length of hospitalization, and 30-day postoperative mortality were documented. Histopathological information was obtained from final pathology reports and included tumor dimensions, pathological TNM stage, the number of harvested lymph nodes per nodal station, and the number of metastatic lymph nodes identified.

### Statistical analysis

Statistical analyses and graphical visualizations were performed using custom Python scripts executed within the Google Colab environment (Google LLC, Mountain View, CA, USA).

Continuous variables were expressed as median and range, depending on data distribution, whereas categorical variables were reported as absolute frequencies and percentages. The distribution of continuous variables was assessed using the Shapiro–Wilk test prior to selecting the appropriate statistical method. Comparisons between groups were performed using the independent samples *t*-test or Mann–Whitney U test for continuous variables and the chi-square test or Fisher’s exact test for categorical variables, as appropriate.

For postoperative morbidity analysis, patients were stratified into two groups based on postoperative evolution: those without clinically relevant morbidity (Clavien–Dindo < II) and those with clinically relevant morbidity (Clavien–Dindo ≥ II).

Univariate logistic regression analysis was initially performed to evaluate potential factors associated with clinically relevant postoperative morbidity. Variables included in the analysis comprised demographic, clinical, functional, and perioperative parameters, including age, sex, CCI, pulmonary function (FEV1), surgical approach, type of resection, and pathological stage.

Variables demonstrating potential clinical relevance or trends toward association in univariate analysis were subsequently included in a multivariate logistic regression model to identify variables associated with increased odds of clinically relevant postoperative morbidity. Odds ratios (OR) and corresponding 95% confidence intervals (CI) were calculated for all regression analyses.

The discriminatory performance of the final multivariate model was evaluated using receiver operating characteristic (ROC) curve analysis. Statistical significance was defined as a two-tailed *P* value < 0.05.

## Results

### Baseline patient characteristics

A total of 163 consecutive patients who underwent curative-intent surgical resection for NSCLC between 2016 and 2023 were included in the final analysis. The mean age of the study population was 61.3 ± 10.2 years. Male patients represented the majority of the cohort (87 cases, 53.4%), while 76 patients (46.6%) were female. Regarding surgical approach, 86 patients (52.8%) underwent open thoracotomy, whereas 77 patients (47.2%) were treated using VATS. Most patients underwent lobectomy (144 cases, 88.3%), while segmentectomy was performed in 19 patients (11.7%). Adenocarcinoma represented the predominant histopathological subtype, accounting for 111 cases (68.1%), while squamous cell carcinoma was identified in 52 patients (31.9%). Early-stage disease was observed in 93 patients (57.1%), whereas 70 patients (42.9%) presented with locally advanced stages. Most patients had a significant comorbidity burden, with 124 (76.1%) having a CCI ≥ 4. Similarly, the majority of patients were classified as ASA III (126 cases, 77.3%). The mean preoperative FEV1 was 80.9 ± 14.7%. Neoadjuvant treatment was administered in 15 patients (9.2%), while documented exposure to respiratory toxic agents was identified in 14 patients (8.6%). Clinically relevant postoperative morbidity (Clavien–Dindo grade ≥ II) was observed in 35 patients (21.5%), whereas 128 patients (78.5%) experienced no postoperative complications or only minor complications that did not require significant therapeutic intervention. A detailed summary of the demographic and clinical data is provided in Table 1.

**Table 1:** Baseline demographic, clinical, and surgical characteristics of the study cohort

Variable	Overall cohort (*n* = 163)
**Age, mean ± SD (years)**	61.3 ± 10.2
**Sex**	
Male	87 (53.4%)
Female	76 (46.6%)
**ASA score**	
II	37 (22.7%)
III	126 (77.3%)
I, IV, V	0 (0%)
**CCI ≥ 4**	124 (76.1%)
**Surgical procedure**	
VATS approach	77 (47.2%)
Open thoracotomy	86 (52.8%)
**Toxic respiratory exposure**	14 (8.6%)
**FEV1, mean ± SD (%)**	80.9 ± 14.7
**Tumor site**	
LLL	30 (18.4%)
LUL	44 (27.0%)
RLL	28 (17.2%)
RML	10 (6.1%)
RUL	51 (31.3%)
**Locally advanced stage**	70 (42.9%)
**Type of resection**	
Lobectomy	144 (88.3%)
Segmentectomy	19 (11.7%)
**History of NAT**	15 (9.2%)

Abbreviations: CCI, Charlson Comorbidity Index; FEV1, forced expiratory volume in one second; ASA, American Society of Anesthesiologists physical status classification; LLL, Left Lower Lobe; LUL, Left Upper Lobe; RLL, Right Lower Lobe; RML, Right Middle Lobe; RUL, Right Upper Lobe; VATS, video-assisted thoracoscopic surgery; NAT, neoadjuvant therapy.

### Comparative analysis between patients with and without clinically relevant postoperative morbidity

Patients were subsequently stratified according to postoperative evolution into two groups: patients without clinically relevant postoperative morbidity (Clavien–Dindo < II) and patients with clinically relevant postoperative morbidity (Clavien–Dindo ≥ II).

Patients who developed clinically relevant postoperative morbidity tended to be older compared with patients without significant postoperative complications (median age 66 vs. 62 years, *P* = 0.0788). Similarly, lower preoperative pulmonary functional reserve was observed in the morbidity-positive group, with lower median FEV1 values compared to patients without clinically relevant morbidity (70% vs. 80%, *P* = 0.0693).

The open surgical approach was more frequently associated with postoperative morbidity, although the difference did not reach statistical significance (*P* = 0.4525). No statistically significant differences were identified regarding pathological stage distribution (*P* = 0.8380), type of resection (*P* = 0.7669), histopathological subtype, neoadjuvant therapy, or exposure to respiratory toxic agents.

The comparative analysis of demographic, clinical, and perioperative variables between patients with and without clinically relevant postoperative morbidity is presented in Table 2.

**Table 2:** Comparative analysis between patients with and without clinically relevant postoperative morbidity

Variable	No morbidity (*n* = 128)	Relevant morbidity (*n* = 35)	Test	*P* value
Age (years)	62 (26–84)	65 (37–81)	Mann–Whitney U	0.0788
FEV1 (%)	80 (55–140)	78 (47–129)	Mann–Whitney U	0.0693
VATS approach	58 (45.3%)	19 (54.3%)	Chi-square	0.4525
Locally advanced stage	56 (43.8%)	14 (40.0%)	Chi-square	0.8380
Segmentectomy	16 (12.5%)	3 (8.6%)	Fisher’s exact	0.7669
Neoadjuvant therapy	12 (9.4%)	3 (8.6%)	Fisher’s exact	1.0000
Squamous histology	44 (34.4%)	8 (22.9%)	Chi-square	0.2753
Male sex	68 (53.1%)	19 (54.3%)	Chi-square	1.0000
CCI ≥ 4	93 (72.7%)	31 (88.6%)	Fisher’s exact	0.0720
ASA III	96 (75.0%)	30 (85.7%)	Chi-square	0.2656
Toxic respiratory exposure	10 (7.8%)	4 (11.4%)	Fisher’s exact	0.5023

Abbreviations: FEV1, forced expiratory volume in one second; VATS, video-assisted thoracoscopic surgery; CCI, Charlson Comorbidity Index; ASA, American Society of Anesthesiologists.

### Univariate logistic regression analysis

Univariate logistic regression was performed to evaluate potential predictors of clinically relevant postoperative morbidity.

Increasing age demonstrated a trend toward association with postoperative morbidity (OR = 1.04, 95% CI, 0.99–1.08; *P* = 0.0657). Similarly, lower FEV1 values demonstrated a tendency toward increased postoperative risk (OR = 0.97, 95% CI, 0.94–1.00; *P* = 0.0991).

Patients undergoing VATS procedures demonstrated comparable morbidity rates relative to open surgery (OR = 1.43, 95% CI, 0.68–3.03, *P* = 0.3473). Advanced pathological stage and type of resection were not significantly associated with clinically relevant postoperative morbidity in univariate analysis.

The results of the univariate logistic regression analysis evaluating factors associated with clinically relevant postoperative morbidity are presented in Table 3.

**Table 3:** Univariate logistic regression analysis for clinically relevant postoperative morbidity

Variable	OR	95% CI	*P* value
Age	1.04	0.99–1.08	0.0657
FEV1	0.97	0.94–1.00	0.0991
VATS approach	1.43	0.68–3.04	0.3473
Locally advanced stage	0.86	0.40–1.83	0.6914
Segmentectomy	0.66	0.18–2.39	0.5235
Neoadjuvant therapy	0.91	0.24–3.41	0.8842
Squamous histology	0.57	0.24–1.35	0.1989
Male sex	1.05	0.49–2.22	0.9029
CCI ≥ 4	2.92	0.96–8.86	0.0591
ASA III	2.00	0.72–5.59	0.1862
Toxic respiratory exposure	1.52	0.45–5.18	0.5012

Abbreviations: OR, odds ratio; CI, confidence interval; FEV1, forced expiratory volume in one second; VATS, video-assisted thoracoscopic surgery; CCI, Charlson Comorbidity Index; ASA, American Society of Anesthesiologists.

### Multivariate logistic regression analysis

Variables considered clinically relevant were subsequently included in the multivariate logistic regression model, including surgical approach, Charlson Comorbidity Index, FEV1, and patient age.

Multivariate analysis demonstrated that an elevated Charlson Comorbidity Index was associated with increased odds of clinically relevant postoperative morbidity (adjusted OR = 2.04, 95% CI, 0.54–7.70; *P* = 0.2948). The VATS approach demonstrated comparable postoperative morbidity risk compared with open surgery (adjusted OR = 1.50, 95% CI, 0.69–3.24; *P* = 0.3044).

Lower preoperative FEV1 values remained associated with increased postoperative risk (adjusted OR = 0.98, 95% CI, 0.95–1.01; *P* = 0.1859), while increasing age demonstrated a modest association with clinically relevant postoperative morbidity (adjusted OR = 1.02, 95% CI, 0.97–1.07; *P* = 0.4618).

**Figure 1:**
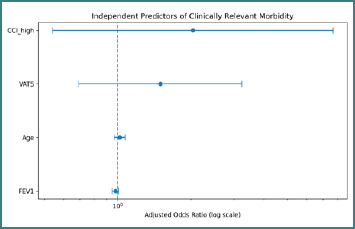
Forest plot of variables associated with clinically relevant postoperative morbidity

The results of the final multivariate logistic regression model are summarized in Table 4 and illustrated in Figure 1.

**Table 4:** Multivariate logistic regression analysis for clinically relevant postoperative morbidity

Variable	Adjusted OR	95% CI	*P* value
VATS approach	1.50	0.69–3.24	0.3044
CCI ≥ 4	2.04	0.54–7.70	0.2948
FEV1	0.98	0.95–1.01	0.1859
Age	1.02	0.97–1.07	0.4618

Abbreviations: OR, odds ratio; CI, confidence interval; VATS, video-assisted thoracoscopic surgery; CCI, Charlson Comorbidity Index; FEV1, forced expiratory volume in one second.

### Predictive model performance

The discriminatory performance of the final multivariate model was evaluated using ROC curve analysis.

**Figure 2:**
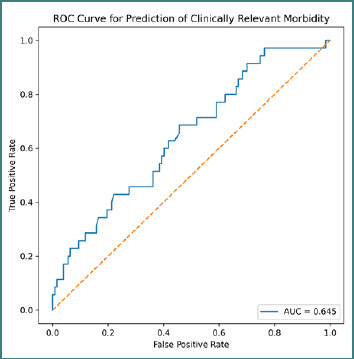
ROC curve analysis of the multivariate predictive model for clinically relevant postoperative morbidity

ROC analysis demonstrated fair predictive performance for clinically relevant postoperative morbidity, with an area under the curve (AUC) of 0.645 (Figure 2).

**Figure 3:**
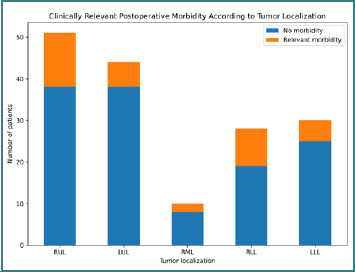
Distribution of clinically relevant postoperative morbidity according to tumor localization. Blue bars represent patients without clinically relevant postoperative morbidity, while orange bars represent patients with Clavien–Dindo grade ≥ II complications. **Abbreviations: LLL - Left Lower Lobe; LUL - Left Upper Lobe; RLL - Right Lower Lobe; RML - Right Middle Lobe; RUL – Right Upper Lobe**.

Tumor localization was additionally evaluated as a secondary exploratory factor potentially associated with clinically relevant postoperative morbidity. No statistically significant association was identified between lobar tumor localization and postoperative morbidity (chi-square test, *P* = 0.354). Nevertheless, right-sided resections—particularly upper- and lower-lobe procedures—tended to show higher postoperative complication rates (Figure 3).

## Discussion

Postoperative morbidity remains a major concern following surgical treatment for NSCLC, despite continuous advances in minimally invasive surgery, perioperative care, and patient selection strategies. Identifying factors associated with clinically relevant postoperative complications is essential for perioperative risk stratification and optimization of surgical management in thoracic oncology patients.

In the present retrospective single-center study, clinically relevant postoperative morbidity (Clavien–Dindo ≥ II) was observed in 21.5% of patients undergoing curative-intent pulmonary resection for NSCLC. Although the predictive performance of the final multivariate model was modest, several clinically meaningful trends were identified, particularly regarding increased comorbidity burden, advanced age, lower pulmonary reserve, and surgical approach.

Comorbidity burden, quantified using the Charlson Comorbidity Index (CCI), was associated with increased postoperative morbidity risk in our cohort. This finding is consistent with previous studies reporting that elevated comorbidity burden negatively influences perioperative outcomes following major pulmonary resection [10,11]. Patients with significant cardiovascular, respiratory, or metabolic comorbidities may present reduced physiological reserve and impaired postoperative recovery capacity, which may contribute to increased postoperative complication rates.

Pulmonary functional reserve also appeared to influence postoperative outcomes in the current study. Lower preoperative FEV1 values were associated with a tendency toward increased postoperative morbidity, supporting the well-established role of pulmonary function assessment in thoracic surgical planning [8,12]. Previous studies have shown that reduced respiratory reserve is associated with increased risk of pulmonary complications, prolonged hospitalization, and postoperative respiratory insufficiency following lung cancer surgery [9].

Increasing age was modestly associated with clinically relevant postoperative complications in our cohort. Although age alone should not represent an absolute contraindication for surgery, elderly patients frequently present higher frailty burden, reduced cardiopulmonary reserve, and increased comorbidity prevalence [6]. Similar observations have been reported in previous thoracic surgical series evaluating perioperative morbidity after pulmonary resection [16].

The role of minimally invasive surgery in reducing postoperative morbidity has been extensively investigated during the last two decades. Multiple studies have demonstrated that VATS is associated with lower postoperative pain, reduced intraoperative blood loss, shorter hospitalization, and faster postoperative recovery compared with conventional thoracotomy [4–6]. In the present study, VATS demonstrated postoperative morbidity rates comparable to those of open surgery, although the difference did not reach statistical significance. This finding may be partially explained by the retrospective nature of the study and by the selection of technically more demanding cases for thoracotomy, including centrally located tumors or cases with increased anatomical complexity.

The predictive performance of the final multivariate model was fair, with an AUC of 0.645. Although the model cannot be considered highly predictive, its performance remains acceptable for an exploratory retrospective cohort study with a relatively limited number of clinically relevant morbidity events. Similar predictive performance has been reported in previous retrospective studies of thoracic oncology evaluating perioperative risk factors [10].

An exploratory analysis evaluating tumor localization suggested a tendency toward increased postoperative morbidity following right-sided resections, particularly involving the right upper and lower lobes, concordant with existing literature [17]. Although statistical significance was not reached, this observation may reflect the greater anatomical variability frequently encountered during right-sided pulmonary resections. However, this result should be verified, and larger studies would be needed to further investigate this potential association.

### Strengths and limitations

An important strength of the present study consists of the relatively homogeneous single-center cohort and standardized perioperative management protocols within a tertiary oncologic institution. Furthermore, the study reflects real-world thoracic surgical practice in an Eastern European oncologic center, a setting that remains comparatively underrepresented in the current literature.

Nevertheless, several limitations must be acknowledged. First, the retrospective and single-center design may introduce selection bias and limit external validity. Second, the relatively limited number of clinically relevant postoperative morbidity events restricted the complexity of the multivariate model and may have reduced statistical power. Third, the non-randomized selection of surgical approach introduces potential confounding factors related to case complexity and surgeon preference. Finally, long-term oncologic outcomes and quality-of-life metrics were not evaluated within the present analysis.

Despite these limitations, the present study suggests that increased comorbidity burden, reduced pulmonary reserve, and advanced age may contribute to clinically relevant postoperative morbidity following NSCLC surgery. Careful patient selection and perioperative optimization remain essential components of thoracic oncologic surgical management.

## Conclusion

Clinically relevant postoperative morbidity remains an important concern following curative-intent surgical treatment for non-small cell lung cancer. In the present single-center retrospective cohort, increased comorbidity burden, advanced age, reduced pulmonary reserve, and surgical approach demonstrated associations with postoperative complication risk.

Although the predictive performance of the final multivariate model was modest, the present findings highlight the importance of careful preoperative patient assessment and individualized perioperative risk stratification in thoracic oncology. Minimally invasive surgical approaches such as VATS may contribute to favorable postoperative outcomes in appropriately selected patients.

Further prospective multicenter studies with larger patient cohorts are required to validate these findings and improve predictive models for postoperative morbidity following NSCLC surgery.

## Data Availability

Data is available upon reasonable request to C.E.
